# P-1080. Multidrug-Resistant Organism Status and Its Association With Hospice Use and End-of-Life Care Patterns in Patients With Advanced Cancer referred Palliative Care

**DOI:** 10.1093/ofid/ofaf695.1275

**Published:** 2026-01-11

**Authors:** Jeong-Han Kim, Jiwon Yu, Ye Sul Jeung, Shin Hye Yoo, Jin-ah Sim, Bhumsuk Keam

**Affiliations:** Ewha Woman University College of Medicine Mokdong Hospital, Department of Internal Medicine, Yangcheon-gu, Seoul-t'ukpyolsi, Republic of Korea; Seoul National University College of Medicine, 2Department of Biomedical Sciences, Jongno-gu, Seoul-t'ukpyolsi, Republic of Korea; Seoul National University Hospital, Center for Palliative Care and Clinical Ethics, Jongno-gu, Seoul-t'ukpyolsi, Republic of Korea; Seoul National University Hospital, Center for Palliative Care and Clinical Ethics, Jongno-gu, Seoul-t'ukpyolsi, Republic of Korea; Hallym University, Department of AI Convergene, Chencheon, Kangwon-do, Republic of Korea; Seoul National University Hospital, Department of Internal Medicine, Jongno-gu, Seoul-t'ukpyolsi, Republic of Korea

## Abstract

**Background:**

Multidrug-resistant organisms (MDRO) are increasingly prevalent and may contribute to more aggressive healthcare utilization near the end-of-life, particularly among patients with advanced cancer receiving palliative care (PC).

**Figure:**
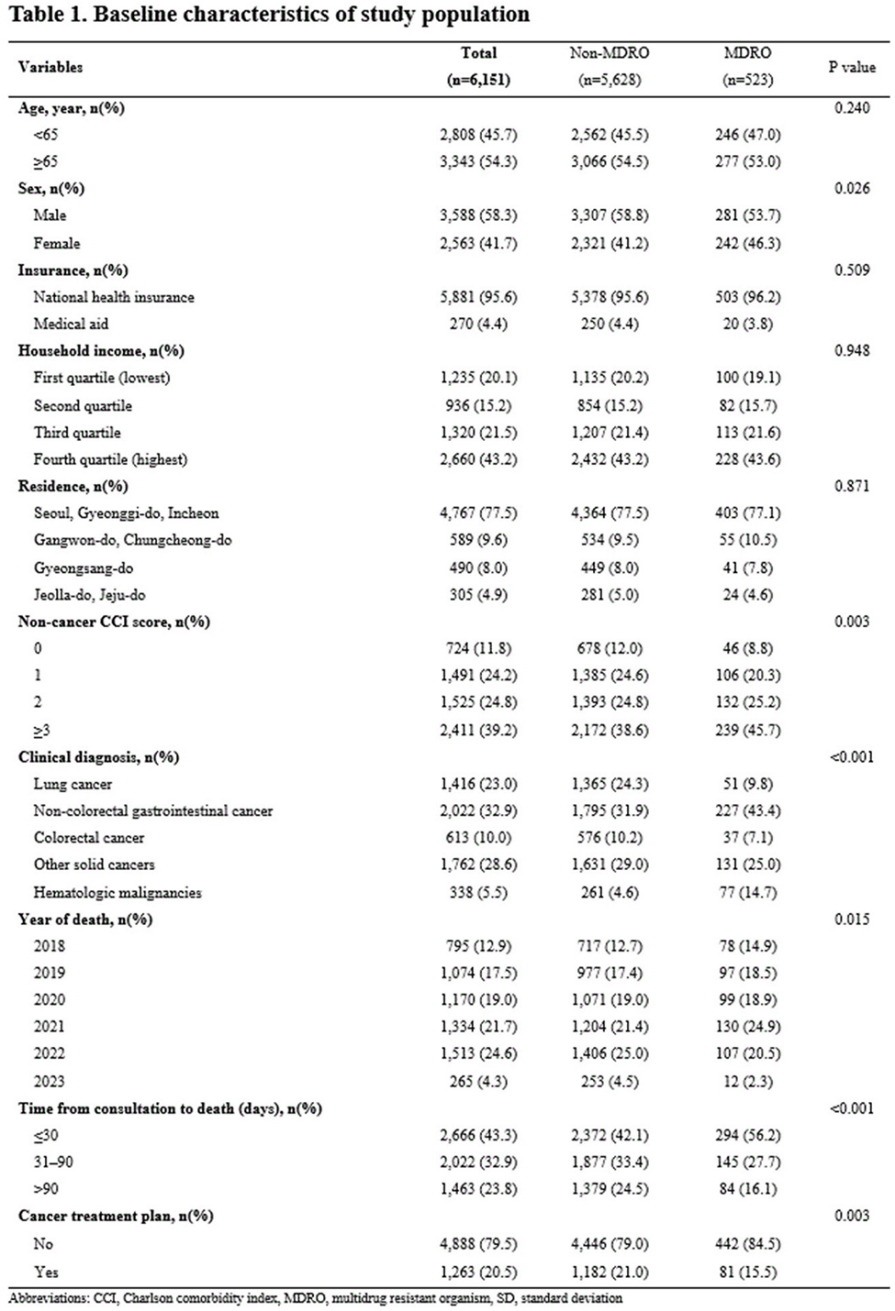
Baseline characteristics of study population

**Figure:**
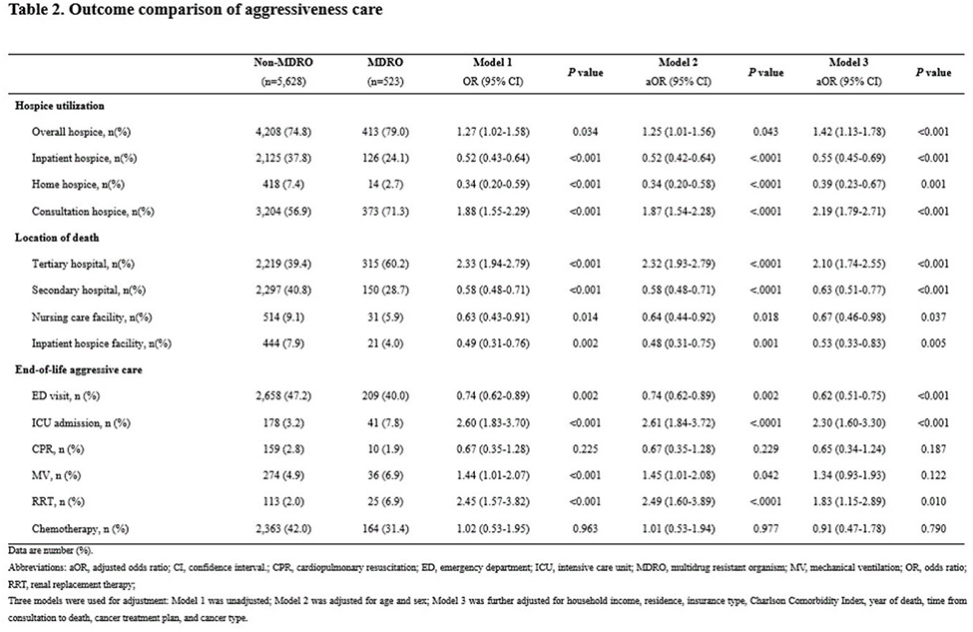
Outcome comparison of aggressiveness care

**Methods:**

We conducted a retrospective cohort study of patients with advanced cancer who received PC consultation at a tertiary hospital in South Korea between January 2018 and December 2022 and died by June 2023. Clinical data were linked to nationwide health insurance claims from the National Health Insurance Service to ensure comprehensive outcome capture. MDRO status was defined by detection of resistant organisms from any clinical specimen. Outcomes included hospice utilization, location of death, end-of-life aggressive care, and medical costs. Adjusted associations were estimated using logistic and gamma regression models.

**Figure:**
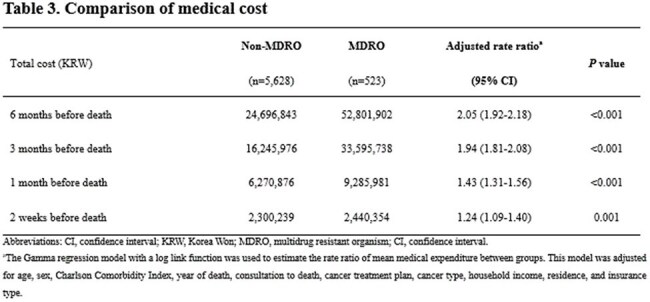
Comparison of medical cost

**Results:**

Among 6,151 patients, 523 (8.5%) had MDRO detected. MDRO status was associated with significantly lower use of community-based hospice care, including inpatient hospice (24.1% vs. 37.8%; adjusted odd ratios [aOR] 0.55, 95% confidence interval [CI], 0.45–0.69; *P*< 0.001) and home hospice (2.7% vs. 7.4%; aOR 0.39, 95% CI, 0.23–0.67; *P*= 0.001). It was also linked to more frequent deaths in tertiary hospitals (aOR 1.97; 95% CI, 1.62–2.39, *P*< 0.001), and higher intensive care unit admissions (aOR 2.18; 95% CI, 1.51–3.16, *P*< 0.001) and renal replacement therapy (aOR 1.63; 95% CI, 1.03–2.60, *P*=0.010). Medical costs were consistently higher in the MDRO group across all end-of-life trajectory before death.

**Conclusion:**

MDRO status may be associated with greater healthcare intensity and the end-of-life aggressive care among patients with advanced cancer referred PC.

**Disclosures:**

All Authors: No reported disclosures

